# Scapular Retraction under Adduction Load: An Alternative to Overhead Exercises to Activate Infraspinatus, Upper, and Lower Trapezius in Subjects with and without Shoulder Pain

**DOI:** 10.3390/ijerph18179251

**Published:** 2021-09-02

**Authors:** Jefferson James dos Santos, Rebeca Orozco Nagy, Matheus Almeida Souza, Leonardo Intelangelo, Michelle Almeida Barbosa, Gabriela Silveira-Nunes, Alexandre Carvalho Barbosa

**Affiliations:** 1 Musculoskeletal Research Group—NIME, Department of Physical Therapy, Federal University of Juiz de Fora, Governador Valadares 35010-180, Brazil; jeffersinhozao@bol.com.br (J.J.d.S.); beckyorozco1996@gmail.com (R.O.N.); malmeida_1812@hotmail.com (M.A.S.); michellecsalmeida@yahoo.com.br (M.A.B.); 2 Musculoskeletal Research Unit—UIM, Department of Physical Therapy, University Center for Assistance, Teaching and Research—CUADI, Universidad del Gran Rosario—UGR, Rosario C1021AAH, Argentina; leonardo.intelangelo@gmail.com; 3 Musculoskeletal Research Group—NIME, Department of Medicine, Federal University of Juiz de Fora, Governador Valadares 35010-180, Brazil; gabrielanunes84@gmail.com

**Keywords:** shoulder impingement syndrome, scapula, exercise therapy, exercise movement techniques, musculoskeletal pain

## Abstract

Exercises for lower trapezius (LT) often use overhead positions, causing compressive forces to the subacromial space. Scapular retraction would be an alternative to activate LT muscle. The present study aimed to assess the excitation levels of infraspinatus, upper trapezius, and lower trapezius muscles during a scapular retraction exercise under progressive adduction loads in subjects with and without painful shoulder. Electromyography of infraspinatus (IS), upper trapezius (UT), and LT was recorded during scapular retraction under progressive adduction loads of 42 participants, divided into two groups: with (SP, *n* = 26) and without shoulder pain (nSP, *n* = 16). The adduction loads of 20%, 30%, 40%, and 50% of the maximal voluntary contraction (MVC) were applied using a load cell. Normalized electromyography and the ratio between UT and LT (UT:LT) were used for statistical analysis. No differences were observed between groups, but a condition effect occurred for all muscles: UT showed higher values at 50% vs. 20% of MVC (*p* = 0.004); LT showed higher values on 40% and 50% of MVC (*p* = 0.001; 0.006). Higher values for IS were noted at 40% of MVC (vs. 20% of MVC; *p* = 0.04) and at 50% of MVC (vs. 20% of MVC; *p* = 0.001, vs. 30% of MVC, *p* = 0.001; vs. 40% of MVC; *p* = 0.001). UT:LT showed lower values at 50% of MVC (vs. 20% of MVC; *p* = 0.001 and vs. 30% of MVC; *p* = 0.016). Scapular retraction with adduction loads at 40–50% is an alternative to overhead exercises aiming to activate the LT and the IS muscles. The exercise ensures higher levels of LT and IS excitation without increasing UT excitation.

## 1. Introduction

Shoulder pain is one of the most common musculoskeletal complaints, affecting 4.7–46.7% of the adult population, and its prevalence increases with age [1,2,3]. Some studies reported a significant association between women and increased prevalence of shoulder pain [4,5]. In fact, a narrative review showed that the estimated prevalence of shoulder pain was higher in women (15.4% in men and 24.9% in women) who reported weekly episodes of pain [6]. Another study retrospectively assessed painful shoulders from medical files, finding a prevalence of women (66.21%) [7]. Additionally, severe pain is more frequently reported in women (56.1%) compared to men (25.0%) [8]. A longitudinal study assessed the risk factors for neck and shoulder pain among young adults in the transition from technical school to working life [9]. The authors reported that high mechanical workload was associated with neck and shoulder pain in women.

A painful shoulder causes mobility restrictions, functional impairments in daily activities, work disability, and a relevant impact on public health services [1,2]. The shoulder pain can last for weeks to months and become a recurrent condition [3,10]. Moreover, aging populations and consequent longer careers might increase the burden of shoulder pain [11], as loss of shoulder muscle mass may impair functionality [12]. Impingement syndrome is a term for injury of structures in the subacromial space [13,14,15]. It has the highest prevalence among shoulder issues, accounting for 36% of shoulder disorders [16,17]. Many shoulder symptoms have been associated with abnormal excitation of the scapular and rotator cuff muscles [18,19]. A systematic review aimed to summarize the current evidence regarding scapulothoracic muscle activity based on surface electromyography in patients with shoulder impingement symptoms or glenohumeral instability vs. healthy controls [20]. The authors concluded that impingement syndrome patients present an overactive upper trapezius and underactive lower trapezius and serratus anterior in contrast to healthy controls. The majority of the included studies showed higher upper trapezius muscle activity during abduction in the scapular plane and in the frontal plane in the impingement syndrome group compared to the control group. For the lower trapezius and serratus anterior, decreased muscle activity was observed in the impingement syndrome group. Prior studies also showed altered movement patterns, including decreased scapular external rotation, increased or decreased scapular upward rotation, and decreased scapular posterior tilting [19,21]. A study reported decreased activity of the infraspinatus muscle in the range of 30°–90° of flexion in the scapular plane, concluding that the deficit in the depression of the humeral head can lead to subacromial friction [22]. It has also been reported that subjects with unilateral subacromial impingement syndrome have decreased infraspinatus muscle activity during external rotation [23].

From a therapeutic perspective, physiotherapy represents one of the primary options for shoulder pain treatment. There is growing evidence of the importance of active exercise therapy in the treatment of shoulder pain [1,3,24]. Optimal exercises for shoulder rehabilitation need to recruit lower trapezius and rotator cuff muscles with concomitant minimal activity of the upper trapezius, as excessive activity of this last muscle might increase the anterior tilt of the scapulothoracic joint [25,26]. Therefore, the shoulder rehabilitation program should include exercises focused on the activation of upward rotators (serratus anterior, upper, and lower trapezius) while minimizing the activation of the scapular downward rotators (latissimus dorsi, levator scapulae, rhomboids, and the pectoralis major and minor muscles) [27] to increase the subacromial space during the overhead movements of the arm and implement effective interventions. A study [28] found a decrease in the electromyographic activity of the anterior deltoid and infraspinatus muscles in 58 individuals with subacromial impingement syndrome between 24 and 85 years with an average of 40 months of pain. Those decreased activities occurred as an adaptive strategy to avoid pain. However, there is no consensus as to the most effective exercise strategy [16,24,29]. Several types of exercises have been proposed, but little data are available to guide the physical therapist in selecting the most appropriate protocol [3,19,24,30,31]. In fact, most of the proposed exercises use an overhead positioning [26,30,32,33]. This position might potentially cause compressive forces to the subacromial space [34,35]. Even in healthy people, overhead exercises such as a wall push-up plus might place the scapula in a position potentially associated with shoulder impingement [36]. Due to shoulder kinematics during a wall push-up plus, such exercise should be cautiously considered during early shoulder rehabilitation, especially in patients with subacromial impingement and/or shoulder pain.

Previous findings showed that subjects with previous shoulder injury have lower activation of the lower trapezius muscle excitation compared to controls and to the contralateral limb [37]. It was originally suggested that global muscle weakness was the main factor for shoulder and scapular problems. However, recent research showed that muscular imbalance might contribute as well [38]. Theoretically, compensation may occur through increased excitation of the upper trapezius muscle combined with decreased excitation and control of the lower trapezius muscle [39]. A study [26] found an increased upper trapezius activity with reduced activation of lower trapezius secondary to shoulder elevation even in retracted scapular position, which leads to consequent higher upper-lower trapezius muscle ratios (UT:LT). This finding suggests the existence of fine activity coordination between the trapezius portions to not counteract the action of the upper trapezius muscle [26]. Thus, an exercise combining low levels of upper trapezius muscle excitation, moderate to high levels of the lower trapezius muscle and rotator cuff muscles excitation, and subacromial non-compressive positioning would be very beneficial for treating subjects with shoulder pain. Accordingly, previous reports and researchers have established a scientific framework that justifies the application of specific exercises in this population.

Therefore, the present study primarily aimed to assess the excitation levels of infraspinatus, upper trapezius, and lower trapezius muscles during a scapular retraction exercise under progressive adduction loads in subjects with and without painful shoulder. A secondary objective was to analyze which load level of resisted adduction would generate the best ratio between upper and lower trapezius muscles. The hypothesis is that the lower trapezius and infraspinatus would present progressive higher levels of muscle excitation during scapular retraction for both groups while the adduction load increases, without any significant increases in upper trapezius muscle excitation nor between-group differences. An additional hypothesis is that the higher levels of adduction loads would generate the desired ratio levels of upper and lower trapezius muscle excitation when compared to lower levels of adduction loads.

## 2. Materials and Methods

### 2.1. Participants

Forty-two subjects participated in this study (Table 1). The subjects were recruited by public invitation through folders and personal contacts. The recruitment proceeded until the sample size was achieved. They were included as they presented themselves for assessment and divided into 2 groups, according to the presence of shoulder pain: with (painful shoulder group) and without shoulder pain (non-painful shoulder group). The subjects were assessed in the biomechanical laboratory facilities on the Federal University of Juiz de Fora—Campus Governador Valadares (Minas Gerais, Governador Valadares, Brazil). All assessments were provided onsite. The objectives of the study were explained to the subjects, and they were notified of the benefits and potential risks involved before signing an informed consent form before participation. The ethics committee for the human investigation of the Federal University of Juiz de Fora approved the procedures employed in the study (protocol number 25614019.4.0000.5147). The G-Power program (version 3.1.5, Franz Faul, Universität Kiel, Kiel, Germany) was used to perform the two-tailed sample calculation using a previous study [27]. The lower trapezius excitation was chosen as the main variable with an effect size of 0.807. The alpha level was set at 5%, and the estimated sampling power was 95%. The software returned a total sample of 22 individuals (*n* = 11 per group) for a sampling power of 0.9504. When considering the maximum drop-out sample margin of 30%, 42 individuals were included in this study for convenience.

Participants were considered healthy and included if they exhibited a full, pain-free range of motion and no symptoms during the physiotherapy assessments. Individuals in both groups were excluded if they had cervical spine-related symptoms, a history of dislocations or shoulder surgery, cortisone injections within the last six months, systemic and neurological reported diseases, adhesive allergies, and history of shoulder fracture or surgery. The subjects were asked to avoid participation in an unusually strenuous activity 48 h before the testing session. All subjects complied with the orientation.

The Shoulder Pain and Disability Index (SPADI—Brazilian Portuguese version) [40] and the quick version of the Disabilities of the Arm, Shoulder, and Hand Questionnaire (QuickDASH—Brazilian Portuguese version) [41] were used to assess the extent of symptoms in the involved shoulder and as additional scores to include and classify the subjects into both groups, according to previously established minimal detectable changes [42,43]. The QuickDASH uses a 5-point Likert scale for answers to questions focused on the level of difficulty of certain tasks, how severe the pain is, and how the disability affects social and work/sport activities. A higher score indicates worse function, with the highest possible score equal to 100. The SPADI was developed to measure current shoulder pain and disability in an outpatient setting. It contains 13 items to assess 2 domains: a 5-item subscale to assess pain and an 8-item subscale that measures disability. The definition of shoulder pain includes recent pain (>1 week) near to anterolateral shoulder region. Patients were included in the SP group if they reported chronic shoulder pain (>1 month during the last year, with a minimum pain intensity of 3/10 on the Numeric Rating Scale) in the anterior deltoid region of their dominant shoulder and if at least 3 of the following criteria were positive: (1) positive Neer sign, designed to reproduce symptoms of rotator cuff impingement through flexing the shoulder and pressure application; (2) positive Hawkins sign, designed to indicate impingement of all structures located between the greater tubercle of the humerus and the coracohumeral ligament. The seated subject flexes the shoulder and the elbow to 90 degrees while the examiner supports proximal to both the patient’s wrist and elbow to ensure maximal relaxation. Then, the examiner rotates the patient’s arm internally; (3) positive Jobe’s sign, which is performed by the examiner passively elevating the patient’s shoulder to 90 degrees of abduction with internal rotation. The examiner then applies downward pressure against the arm. The presence of pain or abnormal weakness is considered positive; (4) painful arc; the patient abducts the arm in the scapular plane. The test is considered positive if the patient experiences pain in the range of 60–120 degrees of abduction, which reduces once past 120 degrees of abduction; and (5) positive resistance test against external rotation, which assesses the infraspinatus and teres minor muscles. The elbow is flexed at 90 degrees and in slight abduction, while the arm is passively externally rotated to its maximum [44].

### 2.2. Instruments

An acquisition module with eight analog channels (Miotec™, Biomedical Equipments, Porto Alegre, RS, Brazil) continuously recorded the biological signals. The conversion from analog to digital signals was performed by an A/D board with a 14-bit resolution input range, the sampling frequency of 2 kHz, a common rejection module greater than 100 dB, signal–noise ratio less than 3 μV Root Mean Square, and impedance of 109 Ω. The collected data were windowed at 125 ms. The sEMG signals were recorded in root mean square in μV with surface Maxicor™ (Maxicor Medical Products, Paraná, Pinhais, Brazil) Ag/AgCl electrodes with a radius of 1 cm and center-to-center distance of 2 cm, applied in a transverse orientation parallel to the underlying fibers on a muscle site. A reference electrode was placed on the left lateral humeral epicondyle. Surface electromyography signals were amplified and filtered (Butterworth fourth-order, 20–450 Hz bandpass filter, 60 Hz notch filter). Before electrode placement, the skin was cleaned with 70% alcohol, followed by an exfoliation using specific sandpaper for skin and a second cleaning with alcohol. The electrodes were positioned on the upper and lower trapezius according to Surface Electromyography for Non-Invasive Assessment of Muscles (SENIAM—http://seniam.org/shoulder_location.htm (accessed on 15 July 2019). SENIAM does not provide electrode placement recommendations for infraspinatus muscle. Therefore, we followed the electrode placement recommendations and descriptions from a previous study [45]. The electrodes were placed on the upper trapezius at 50% on the line from the acromion to the spine on vertebra C7. For the lower trapezius, the electrodes were placed at 2/3 on the line from the trigonum spinea to the 8th thoracic vertebra. Finally, for the infraspinatus muscle, the electrodes were placed 4 cm below the spine of the scapula over the infrascapular fossa of the scapula, laterally, but not over the posterior deltoid muscle.

**Figure 1:**
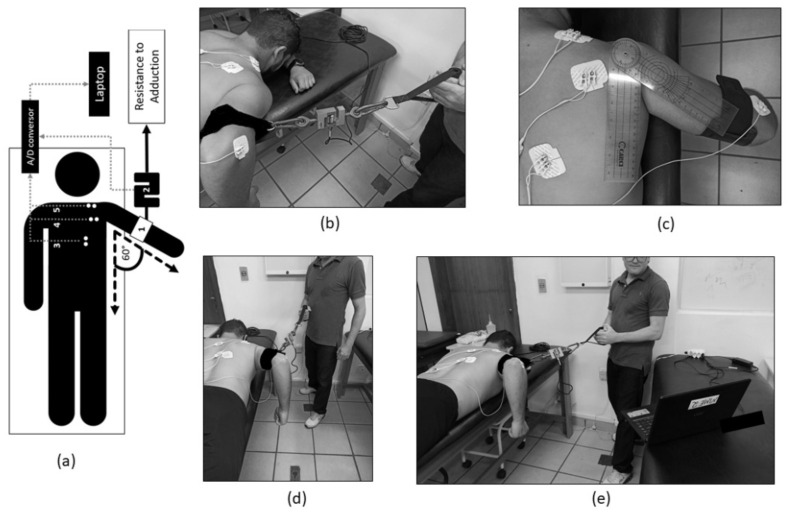
Procedure for data recording. Subject in prone (**a**,**b**), leaving the arm out of bed in 60 degrees of abduction (**c**). The strap positioned right above the humeral epicondyles’ line (**c**). The rater and participant’s initial positioning (**d**). Scapular retraction while the rater controls the level of adduction load using the biofeedback software (**e**). Legend (**a**): 1. strap; 2. load cell; 3. surface electromyography sensor (sEMGs) positioning—lower trapezius; 4. sEMGs positioning—infraspinatus; 5. sEMGs positioning—upper trapezius.

A calibrated load cell (Miotec™, Biomedical Equipments, Porto Alegre, RS, Brazil; maximum tension–compression = 200 kgf, the precision of 0.1 kgf, the maximum error of measurement = 0.33%) was used to control the levels of adduction loads. The validity of the load cell was previously assessed [46]. The load cell was coupled and synchronized with the acquisition module. Two straps were used to handle the load cell. Both were connected to the load cell (Figure 1a). The 1st strap was positioned on the subject’s distal arm (right above the humeral epicondyles’ line). The 2nd strap was used by the rater to control the level of load applied in each condition. All pieces of information were recorded and processed offline using the software Miotec Suite™ (Miotec Biomedical Equipments, Porto Alegre, RS, Brazil).

### 2.3. Maximal Voluntary Isometric Contraction for Electromyography Normalization Purposes

Three 5 s maximal voluntary isometric contractions (with 3 min of rest between each trial) from each muscle were performed by each participant. For each tested muscle, a rest of 5 min was allowed. Standardized verbal commands (“start”, “force”, “stop”) were used by the same rater for all test recordings. Before recording the first maximal voluntary isometric contractions trial, the participants received an explanation about the positioning and the direction they should exert maximal effort. Then, a 5 s familiarization trial followed by 3 min of rest was performed to verify the participants’ understanding of the procedures. The mean among the maximal voluntary isometric contractions trials was used to normalize the electromyographic signals. For the infraspinatus and upper trapezius muscles, the participant remained standing. For the infraspinatus, the shoulder was externally rotated with the elbow flexed at 90°. The participant was requested to perform an isometric contraction of shoulder external rotators against the rater’s manual resistance [45]. For the upper trapezius muscle, the patient resisted abduction with the arm placed in 90 degrees of shoulder abduction; the elbow flexed 90 degrees [47]. For the lower trapezius muscle, the participant was asked to lie in the prone position. The maximal voluntary isometric contraction was collected with the shoulder fully extended with the scapula maximally retracted, while the resistance was applied against the fully flexed elbow on the distal humerus. The participant was instructed to resist against the downward force. The scapular retraction was chosen due to its capacity to activate specific sEMG patterns in the lower trapezius muscle [48]. Participant’s positioning was monitored by the researcher to minimize compensatory movements.

### 2.4. Maximal Voluntary Isometric Contraction to Control the Adduction Resistance

Three 5 s maximal voluntary isometric contractions (with 3 min of rest between trials) in adduction were recorded using the load cell. The subject was instructed to lie prone, leaving the arm to be evaluated out of bed. The therapist positioned the strap on the distal third of each subject’s arm (right above the humeral epicondyles’ line). The subject was instructed to maximally pull the load cell through the attached strap for 5 s. Standardized verbal commands (“start”, “force”, “stop”) were used by the same rater for all tests’ recordings.

### 2.5. Exercise Description

The subject was instructed to lie prone, leaving the arm to be evaluated out of bed parallel to the floor (Figure 1b). All sensors for electromyography were previously positioned. The therapist positioned the strap on the distal third of the subject’s arm right above the line between the humeral epicondyles. Additionally, the magnitudes of progressive resistance were percentages of the individual maximal voluntary isometric contraction in adduction. After 10 s of initial rest, resisted adductions at 60 degrees (measured by goniometer—Figure 1c) were performed for 10 s at 20%, 30%, 40%, and 50% of the previously collected adduction maximal voluntary isometric contraction with 20 s of rest between tasks (Figure 1e). As the participant lay in prone, the rater asked the participant to actively adduct the arm. When the chosen level was achieved, the therapist asked the participant to hold the contraction. Concomitantly, the subject was instructed to perform maximal scapular retraction of the evaluated shoulder (Figure 1e). The level of resistance was applied by the therapist as previously set and real-time controlled using the Biotrainer™ visual biofeedback software (Miotec™, Biomedical Equipment, Porto Alegre, RS, Brazil). The Excel software was used to randomize the trials’ order. To prevent fatigue, only 1 set of trials was performed. To ensure the absence of muscle fatigue, the median frequency was obtained using the real-time feedback software and monitored during the entire protocol. The median frequency from the 50% and the 20% were extracted to check if there were differences between them. All assessments were provided onsite. No practice at home was asked. Attendance at the session was, therefore, taken as compliance with the assessment protocol. No co-interventions were performed in either group, and no adverse effects were reported by any participant during the assessments.

### 2.6. Data Extraction

All data were extracted offline using the Miotec Suite™ Software (Miotec™, Biomedical Equipments, Porto Alegre, RS, Brazil). As the load cell was synchronized with the electromyography channels, the trained rater set the interval using the force onset. Three 1 s windows of the initial rest were collected (first 10 s of rest), and the force onset was defined by three times the standard deviation from the averaged rest intervals plus the mean itself. The interval started when the signal exceeded the onset threshold value. Conversely, the end of the interval was set using the same threshold. The interval means were used for statistical analysis (mean muscle excitation). The ratio between the upper and lower trapezius muscles was also calculated by dividing the normalized electromyographic mean obtained from the upper trapezius by the obtained from the lower trapezius [49].

### 2.7. Statistics

Data were presented as means and standard deviations. The mixed factorial Analysis of covariance (ANCOVA) with repeated measures was used to rate within- and between-group differences. The factor group*condition was also assessed. Co-variates were included to assess the influence of BMI and weight. The condition is defined as the level of adduction load applied during the protocol. All data were reworked using Holm’s post hoc test to compare differences among trials (20%, 30%, 40%, and 50% of maximal adduction contraction), avoiding pairwise multiple comparisons. The significance was set at *p* < 0.05. The standardized differences for the comparisons were analyzed using the partial eta square’s effect size (*η*^2^_p_). The magnitude of the *η*^2^_p_ was qualitatively interpreted using the following thresholds: ~0.01 (small), >0.09 (medium), and >0.25 (large). All analyses were performed using the JAMOVI software (Version 0.9.6, JAMOVI project, 2019).

## 3. Results

Descriptive and inferential data are presented in Table 2. No differences were observed between 50% and 20% median frequency values during the protocol (*p* > 0.05), ensuring no influence of fatigue. No effect was found for any covariate. Both groups presented similar patterns of muscle activity, with low levels of excitation of the upper trapezius muscle. No between-group differences were observed for the upper trapezius muscle (F = 0.39; *p* = 0.537; *η*^2^_p_ = 0.012), the lower trapezius muscle (F = 0.14; *p* = 0.294; *η*^2^_p_ = 0.033), the infraspinatus muscle (F = 1.96; *p* = 0.171; *η*^2^_p_ = 0.058), and for the ratio UT:LT (F = 1.82; *p* = 0.186; *η*^2^_p_ = 0.052).

Progressive higher levels of the lower trapezius and infraspinatus excitation were observed during scapular retraction. A progressive effect for adduction load condition was observed (Table 2) at within-group analysis (upper trapezius: F = 4.19; *p* = 0.008; *η*^2^_p_ = 0.11/lower trapezius: F = 32.7; *p* = 0.001; *η*^2^_p_ = 0.49/infraspinatus: F = 19.09; *p* = 0.001; *η*^2^_p_ = 0.37). The upper trapezius pairwise comparison showed higher values at 50% compared to 20% of maximal adduction load, but always below 20% of the maximal voluntary isometric contraction. For the lower trapezius, higher values were observed at 40% and 50% of the maximal adduction load, with differences as follows: 20% < 40%, 20% < 50%, 30% < 40%, 30% < 50%, and 40% < 50%. For infraspinatus, higher values at 40% and 50% of the maximal adduction load differences were noted as follows: 20% < 40%, 20% < 50%, 30% < 50%, and 40% < 50%.

Higher levels of adduction loads generated lower ratio levels of upper and lower trapezius muscle excitation (Table 2). An effect was observed for UT:LT ratio comparing 20% vs. 50% and 30% vs. 50% (F = 5.83; *p* = 0.001; *η*^2^_p_ = 0.15). The UT:LT ratio at 50% was significantly lower than 20% and 30%.

## 4. Discussion

The primary focus of this study was to investigate the excitation of the infraspinatus, upper trapezius, and lower trapezius muscles during the scapular retraction exercise while progressive adduction loads were controlled by biofeedback. The results showed no differences between SP and nSP groups, but there was an effect among distinct levels of adduction loads. The absence of between-group differences constitutes an important finding, as it suggests the benefits of the current exercise in accomplishing the exercise’s goals for both groups meaning that individuals with shoulder pain were able to achieve the same levels of muscle excitation on each level of adduction load compared to those without pain during the scapular retraction.

A study assessed the excitation levels of the upper and lower trapezius, among other muscles, during shoulder shrugging and retraction exercises [27]. The findings showed low to moderate levels of excitation for the upper trapezius (25–38% of maximal voluntary isometric contraction) with even lower levels for lower trapezius (3–22% of maximal voluntary isometric contraction). Additionally, the exercises were all performed in a potential compressive position. The exercise proposed in the current study would potentially avoid subacromial compression due to non-overhead positioning [25,26], with additional advantages in targeting the lower trapezius muscle excitation concomitant to lower upper trapezius excitation. Such type of exercise would be an option for exercise prescription aiming the success of the therapy [50]. Another study assessed the levels of scapular muscles during scapula retraction exercises at various shoulder abduction angles [25]. The authors reported low levels of upper trapezius excitation (6–18% of maximal voluntary isometric contraction) and moderate middle trapezius recruitment (22–31% of maximal voluntary isometric contraction). However, the lower trapezius muscle was not assessed. Muscle activation below 20% is considered clinically low, and activity between 20% and 40% is considered clinically moderate [48,51]. An important purpose of the exercises for shoulder rehabilitation is to recruit lower trapezius and rotator cuff muscles with concomitant minimal activity of the upper trapezius, as excessive activity of this last muscle might increase the anterior tilt of the scapulothoracic joint [25,26], leading to compensations. To our knowledge, there is no report in the literature of an exercise addressing combined higher excitation of the lower trapezius and infraspinatus muscles without increasing the upper trapezius excitation and potentially minimizing the impact without deteriorating the subacromial space or exacerbating the painful condition. A study assessed the effects of scapular retraction–protraction position with scapular elevation on shoulder girdle muscle activity during glenohumeral abduction [52]. The lower trapezius activity was significantly higher in retracted scapular position than in neutral and protracted positions, both with and without scapular elevation. However, the lower trapezius activity was significantly lower with scapular elevation (i.e., with concomitant upper trapezius muscle contraction) than without during retracted scapular position. The authors suggested the existence of a fine trapezius coordination pattern between the muscle portions to not counteract the action of the upper trapezius muscle and promote smooth scapular movements. We hypothesize the voluntary contraction due to adduction load affected their abductor UT counterpart, prioritizing the LT excitation due to biomechanical moment on the scapular girdle.

The present results suggest a pattern of muscle excitation that, in opposition to upper trapezius recruitment, the shoulder adduction leads to progressive higher values of lower trapezius excitation combined with very low values of upper trapezius muscle activity using the concomitant progressive adduction loads. A study had similar findings using the infraspinatus and the deltoid muscles [53]. The humerus was actively adducted against a sphygmomanometer (using 80% maximum force output) while maximal effort external rotation was manually resisted. The results showed a reduced posterior deltoid electromyographic activity during the active adduction compared with no resisted adduction without reducing the activity of the infraspinatus.

Previous studies showed that moderate levels of muscle activation (20–40% of the maximal voluntary isometric contraction) are considered adequate to retrain neuromuscular control of the scapular girdle [48,54]. As the upper trapezius excitation showed a low level of muscle activation (range between 6% and 9% of maximal voluntary isometric contraction) with moderate to high levels of the lower trapezius excitation (range between 34% and 87% of maximal voluntary isometric contraction), further studies may consider the role of the present exercise in a neuromuscular training protocol. Moreover, the upper trapezius muscle excitation is low enough, and lower trapezius muscle excitation is high enough to potentially cause effects on prospective training, as many authors have proposed exercises that emphasize the activation of the lower trapezius with less activation of the upper trapezius to improve the neuromuscular control of the scapula and to prevent and treat people with shoulder pain [48,55]. Altered patterns of muscle excitation may represent attempts to minimize muscle activity during painful movements. The altered neural control, muscle weakness, and muscle imbalance are factors that account for the reduction in muscle excitation [18,37]. The increased level of lower trapezius activity combined with very low upper trapezius recruitment found in the present exercise may minimize the compensatory upper trapezius muscle activity usually found in patients with a painful shoulder [26]. Furthermore, the high achieved levels of lower trapezius muscle excitation can be potentially useful to contribute to strength and coordination training. In an interesting study, the activity levels of the upper trapezius, middle trapezius, and lower trapezius muscles were measured during various tasks and selectively activated each of the muscles [56]. The authors found that the patients with shoulder impingement syndrome were unable to activate middle trapezius and lower trapezius muscle as highly as healthy individuals. Another study investigated differences in muscle excitation by comparing previously injured limbs of individuals with a history of glenohumeral joint injury and healthy matched controls during functional isometric contractions [37]. In the previously injured group, the involved lower trapezius muscle was significantly less activated than the same muscle of the uninvolved limb during scaption and abduction.

The UT:LT ratio results showed progressively lower values at 40% and 50% of maximal adduction load compared to 20% and 30%. Lower values of UT:LT ratio indicate the desirable scenario with higher lower trapezius excitation combined with lower upper trapezius excitation. The UT:LT ratio has been considered an important factor as the scapular muscles do not work in isolation but synergistically to produce a controlled scapular movement with consequent shoulder stability [25,56,57]. Previous studies have assessed scapular muscle excitation and reported a higher UT:LT ratio in those with shoulder impingement syndrome compared with healthy controls [25,26]. The scapular motion may be disrupted during dynamic arm activities when the activity between the scapulothoracic muscles is not coordinated [26,49]. A study showed a disruption in muscle activity ratios between sections of the trapezius and between the trapezius and serratus anterior during an active arm elevation task in patients with subacromial pain syndrome, highlighting the lower trapezius muscle as an important part of altered ratios during arm ascent and descent [49].

In terms of practical applications, the healthy controls and subjects with shoulder pain showed similar upper trapezius, lower trapezius, and infraspinatus muscle excitation during scapular retraction exercise with progressive adduction loads, suggesting that the exercise could be potentially used in strengthening programs without leading abnormal trapezius muscle activity in subjects with shoulder pain. An important topic is that no load was applied specifically against the retraction movement but only counteracting the adduction. Thus, a greater level of excitation could be achieved in all scapular muscle retractors by directing loads in other planes. However, further prospective studies need to clarify the utility of the exercise in a rehabilitation training program. Furthermore, a distinct study design may elucidate the level of muscle excitation with additional resistance applied to scapular retraction.

Limitations of the present study must be addressed. Distinct shoulder angles may elicit different levels of excitation for all muscles, as the present study did not assess the shoulder full range of motion. While this study provided useful information regarding the muscles being activated during various levels of adduction loads, no scapular kinematics was documented due to technical issues (e.g., absence of 3D analysis), as the laboratory did not have a system to acquire 3D movement data. Additional information about scapular movements, along with the others scapular girdle and rotator cuff muscles excitation, would provide data to select exercises based on the subject’s needs. Another important limitation is the limited number of muscles assessed by the present study. Other muscle interactions may be elicited with complementary electromyographic analysis. The convenience sample was also constituted by young adults and mostly by women. The results may vary in different ranges of age, as well as in other gender proportions.

## 5. Conclusions

The present results support the use of 40–50% adduction loads to ensure higher levels of lower trapezius and infraspinatus excitation without increasing upper trapezius muscle excitation. The scapular retraction exercise under 40 and 50% of maximal voluntary isometric contraction adduction loads is an option to ensure increased levels of excitation of the infraspinatus and lower trapezius with low levels of upper trapezius muscle excitation, which is an alternative to overhead exercises aiming to activate the lower trapezius and the infraspinatus muscles.

## Figures and Tables

**Table 1:** Participants’ characteristics.

Outcome	SP Group	nSP Group	*p*
*n* (male/female)	26 (7/19)	16 (5/11)	0.630 ^a^
Age (years)	26 ± 8	22 ± 2	0.138 ^b^
Weight (kg)	67 ± 12	57 ± 10	0.018 ^b^
Height (m)	1.69 ± 8	1.66 ± 10	0.286 ^b^
BMI (kg/m^2^)	23 ± 5	21 ± 5	0.036 ^b^
Dyskinesis (yes/no [%])	9/91	7/93	0.837 ^a^
QuickDASH score	25 ± 3	2 ± 1.7	>0.001 ^c^
SPADI-total	31 ± 16	2.4 ± 0.5	>0.001 ^c^
SPADI-pain	40 ± 4.4	3.6 ± 1.9	>0.001 ^c^

Legend: SP—with shoulder pain; nSP—without shoulder pain; ^a^ Chi-square test; ^b^ Student’s independent *t*-test; ^c^ Mann–Whitney test.

**Table 2:** Muscle excitation at different adduction loads (in % of the maximal voluntary isometric contraction using the load cell).

Muscle	Adduction Load Condition	SP Group (%)	nSP Group (%)	Between-Group (F; *p*-Value)	Within-Group Pairwise Comparison for Condition Effect (*p*-Value)
Upper Trapezius	20%	6 ± 4	6 ± 5	0.39; 0.537	20% < 50% (*p* = 0.004)
	30%	8 ± 7	6 ± 5		
	40%	7 ± 6	6 ± 3		
	50%	9 ± 7	7 ± 4		
Lower Trapezius	20%	34 ± 16	49 ± 29	1.14; 0.294	20% < 40% (*p* = 0.001)
	30%	46 ± 26	54 ± 35		20% < 50% (*p* = 0.001)
	40%	58 ± 40	66 ± 37		30% < 40% (*p* = 0.006)
	50%	72 ± 44	87 ± 42		30% < 50% (*p* = 0.001)
Infraspinatus	20%	23 ± 14	18 ± 6	1.96; 0.171	20% < 40% (*p* = 0.040)
	30%	25 ± 17	17 ± 6		20% < 50% (*p* = 0.001)
	40%	29 ± 21	21 ± 6		30% < 50% (*p* = 0.001)
	50%	37 ± 27	27 ± 8		40% < 50% (*p* = 0.001)
UT:LT	20%	0.22 ± 0.18	0.16 ± 0.13	1.82; 0.186	20% < 50% (*p* = 0.001) 30% < 50% (*p* = 0.016)
	30%	0.22 ± 0.20	0.14 ± 0.08		
	40%	0.18 ± 0.15	0.13 ± 0.11		
	50%	0.16 ± 0.14	0.10 ± 0.06		

Legend: SP—with shoulder pain; nSP—without shoulder pain; UT:LT—ratio between upper and lower trapezius muscles.

## Data Availability

The data presented in this study are openly available in Barbosa, Alexandre (2021), “Scapular retraction under adduction load: an alternative to overhead exercises to activate infraspinatus, upper, and lower trapezius in subjects with and without shoulder pain”, Mendeley Data, V1, doi:10.17632/2sv5hbdz4s.1.
